# Role of the *ACE* I/D Polymorphism in Selected Public Health-Associated Sporting Modalities: An Updated Systematic Review and Meta-Analysis

**DOI:** 10.3390/ijerph21111439

**Published:** 2024-10-29

**Authors:** Lydia Sommers, Liz Akam, David John Hunter, Jasvinder Singh Bhatti, Sarabjit Mastana

**Affiliations:** 1 School of Sport, Exercise and Health Sciences, Loughborough University, Loughborough LE11 3TU, UK; lydiasommers4@gmail.com (L.S.); e.c.akam@lboro.ac.uk (L.A.); d.j.hunter@lboro.ac.uk (D.J.H.); 2 Department of Human Genetics and Molecular Medicine, Central University of Punjab, Bhatinda 151401, India; jasvinder.bhatti@cup.edu.in

**Keywords:** *ACE* I/D polymorphism, meta-analysis, endurance, power athletes

## Abstract

Background: The *ACE* I/D polymorphism has been suggested to be associated with multiple chronic diseases and sports modalities, which has public health implications for global populations and sport performance. This updated review aims to strengthen the association and identify sporting disciplines that are most influenced by the *ACE* gene polymorphism using a meta-analysis approach. Methods: Published studies on the association between the *ACE* I/D polymorphism and elite endurance and power were collected until 15 June 2024. The studies on public health-associated sports like running, swimming, and cycling were systematically reviewed following pre-agreed criteria, and a meta-analysis was carried out using different genetic models. Results: A total of 137 studies were identified in the literature search and screened. There was a significant association between elite endurance and the *ACE* II genotype compared with healthy inactive controls (OR, 1.54; 95%CI, 1.24–1.91) and elite power athletes (OR = 1.56; 95%CI = 1.07–2.28). Specifically, runners and triathletes were associated with the II genotype compared with controls (OR = 1.76; 95%CI = 1.26–2.47; *p*-value = 0.001 and OR = 2.69; 95%CI = 1.15–6.32, *p*-value = 0.023, respectively). Additionally, endurance swimmers were associated with the II genotype compared with short-distance, power swimmers (OR = 2.27; 95%CI = 1.49–3.45; *p*-value < 0.001). Conclusion: The meta-analysis results confirm and strengthen the association between elite endurance and the *ACE* I/D polymorphism in different sporting modalities, which may have implications for public health and sports participation.

## 1. Introduction

Sports performance, especially elite endurance performance, is a complex phenotype characterised by the ability to sustain high workloads for extended periods. Many physiological characteristics influence endurance, including high maximal oxygen uptake, high haemoglobin, metabolic efficiency, oxidative enzyme profile, skeletal muscle fibre composition, and biomechanics [1]. In addition, environmental factors such as living at altitude, diet, and motivation also contribute and are often impacted by culture, society, public interest, participation, and accessibility [2,3]. The concept that sports performance relies on gene variants that directly impact molecular, cellular, and behaviour sport-related phenotypes is widely accepted [4]. However, this area remains poorly understood and arguably one of the most ambiguous contributors because of conflicting studies.

Advances in genetic research over the last decade have allowed for a better understanding of the role of certain DNA polymorphisms in influencing endurance performance. Additive genetic factors account for approximately 66% of the variance in athlete status [5,6]. This variance may explain why only some athletes reach elite standards and, among this elite population, why a few go on to achieve world records or multiple international championship titles. Determining precisely which variants are responsible and the underlying mechanisms involved has been the focus of research for the last 30 years. Exercise phenotypes are polygenic [7]; to date, over 251 DNA variants have been associated with athlete status [8]. The elite phenotype is likely the result of an additive genetic effect stemming from a precise combination of DNA variants. To date, 41 endurance-related, 45 power-related, and 42 strength-related DNA variants have been positively associated with athlete status in at least two studies [8]. As research continues to identify these variants and their interactions, the complexity of genetic contributions to elite performance becomes increasingly evident, underscoring the need for further investigation.

Extensive investigations have focused on the angiotensin-converting enzyme (*ACE*) I/D polymorphism (rs4646994) and *ACTN3* R/X gene polymorphisms [8,9,10,11,12]. The *ACE* I/D polymorphism (rs4646994) is arguably the most studied gene variant [4,13,14]; however, the precise weight and underlying mechanisms of the effect of *ACE* I/D on the elite endurance phenotype and the specific sports associated with it remain unclear.

The *ACE* gene is on chromosome 17 in position 17q23.3 and plays a vital role in the renin–angiotensin system (RAS), thereby regulating blood pressure by converting angiotensin I into angiotensin II (ANG II). ANG II is a potent vasoconstrictor [15]; it increases blood pressure by stimulating aldosterone, causing renal reabsorption of sodium and water to increase blood volume while simultaneously degrading bradykinin, a vasodilator [16]. Additionally, ANG II has a trophic effect on endothelial, skeletal, and cardiac muscle cells [16,17] by stimulating growth factors that induce cell proliferation [18]. Consequently, there is a well-established connection between *ACE* and myocardial infarction, coronary heart disease, and ischemic and idiopathic cardiomyopathy [19,20,21]. This connection explains the widespread clinical use of ACE inhibitors. Local RASs exist in many tissues, including skeletal muscle, adipose and myocardium [22,23]. Given that endurance exercise relies on the prolonged optimal function of these tissues, it is posited that ACE influences athletic performance, particularly in elite endurance contexts.

The *ACE* insertion/deletion (I/D) (rs4646994) polymorphism refers to the presence or absence of a 287bp sequence in intron 16 of the *ACE* gene. *ACE* I/D accounts for up to 47% of the ACE activity variance in subjects and has an additive effect across genotypes II, ID, and DD [24]. In addition, there is a significant association between the *ACE* I* allele and reduced serum and tissue ACE activity compared with the D* allele [24,25], as well as a significant association between the D* allele and increased degradation of bradykinin [26]. This suggests that individuals carrying the I* allele may experience enhanced vascular function, improving blood flow and oxygen delivery to the muscles during prolonged physical activity, ultimately contributing to superior endurance performance. This highlights the potential role of genetic variation in influencing endurance capacity and athletic success in elite populations. Furthermore, previous studies suggest that ethnicity may influence the *ACE* I/D polymorphism and its relationship with serum ACE activity. Gainer et al. [27] reported that black individuals heterozygous for the *ACE* I/D polymorphism had a markedly reduced response to bradykinin-induced vasodilation compared with white individuals. Additionally, Barley et al. [28] reported that there was a higher frequency of the D* allele in Nigerians compared with a higher frequency of the I* allele in the Samoans.

This updated systematic review and meta-analysis aims to determine whether the *ACE* I/D (rs4646994) polymorphism influences performance across different public health-associated endurance/sporting modalities and to elucidate the specific mechanisms at play. While substantial research exists, significant gaps and inconsistencies persist regarding the precise impact and underlying mechanisms of the *ACE* I/D polymorphism on the elite endurance phenotype, particularly in specific sports. This review seeks to aggregate and synthesise existing studies, providing a comprehensive understanding of the current state of knowledge while identifying trends and patterns that emerge from the literature.

## 2. Methods

### 2.1. Eligibility Criteria

This review used Richardson et al.’s [29] PICO-style approach to characterise the eligibility criteria (population, intervention, comparison, outcome) using PRISMA 2020 guidelines [30].

#### 2.1.1. Population

Observational studies of adult (18–40 years) elite endurance athletes from individual sporting disciplines were included. No restrictions on ethnicity were applied. This review identified elite athletes as individuals who have reached international level competition (competed for their country). This standard requires high event speciality and training volume to ensure differentiation from the average person. This review excluded studies that (1) did not define the standard of athletes, (2) did not mention the sporting disciplines involved, (3) involved elite athletes under 18 years or above 40 years, and (4) only used power or team sport athletes. Excluding young elite athletes eliminated concerns over underdevelopment and lack of event speciality. Removal of studies measuring team sports ensured that endurance remained the predominant measure. Studies that involved a mixture of individual and team sports were used if they were analysed separately so that the team sport data could be excluded. This review excluded animal studies and those that focused on disease prevention.

#### 2.1.2. Intervention

Interventions not deemed eligible were those that (1) focused on disease prevention, (2) focussed on an *ACE* variant other than the *ACE* I/D polymorphism (rs4646994), (3) studied sports injuries, (4) incorporated abnormal environmental conditions such as high altitude, and (5) did not measure endurance athletes.

#### 2.1.3. Comparison

This review considered endurance/power comparisons and endurance/control comparisons. Therefore, studies involving an elite power population in addition to or instead of a healthy control population were included in the analysis via endurance/power comparisons. Ethnicity-matching between the controls/elite power athletes and the endurance athletes was essential to prevent racial skew. Furthermore, controls had to be healthy and inactive for accurate comparisons to elite endurance athletes. Studies that conducted comparisons with endurance athletes (i.e., not with controls or power athletes) were ineligible.

#### 2.1.4. Outcome

The primary outcome measure was the incidence of the I* allele among endurance athletes compared to controls or power athletes. Therefore, the included studies had to provide distributions of all possible genotypes (II, ID, and DD) for endurance athletes and controls or power athletes, as well as a clear outcome relating to the *ACE* I/D polymorphism and endurance athletes. For example, studies that focused only on the outcome of power athletes were excluded. Deviation from Hardy–Weinberg equilibrium (HWE) prompted exclusion.

### 2.2. Literature Identification

**Figure 1:**
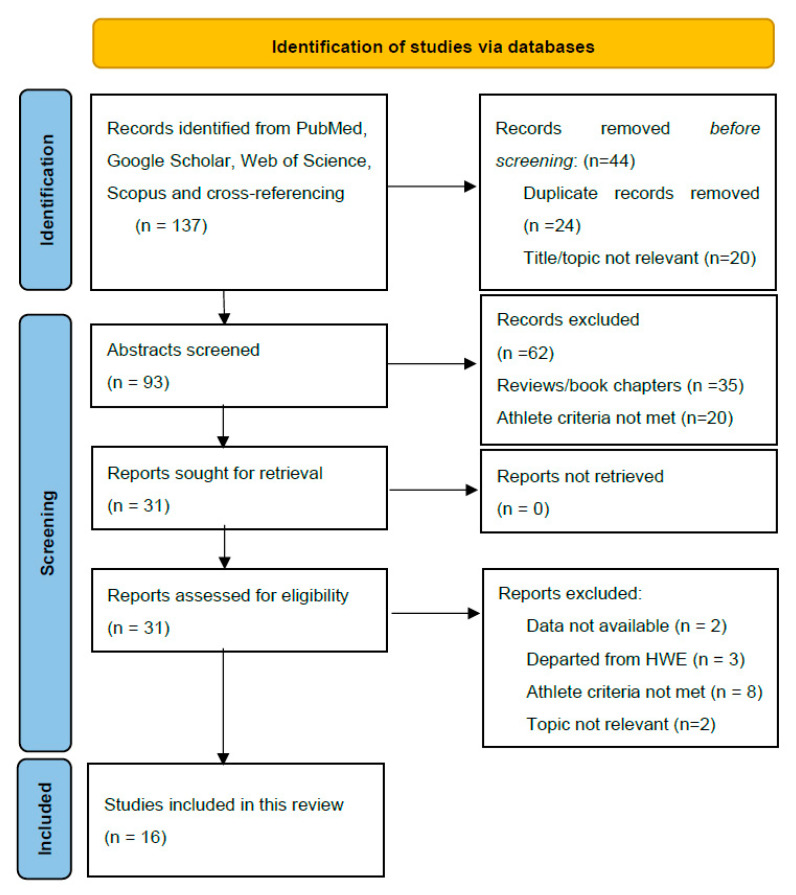
PRISMA 2020 flow diagram used in this systematic review.

PubMed, Web of Science (WoS), Scopus, and Google Scholar were used to identify studies addressing the effects of the *ACE* I/D polymorphism on endurance performance until 15 June 2024. The search identified a total of 137 articles for screening. Figure 1 displays the screening process. Combinations of essential criteria such as “*ACE* I/D polymorphism”, “angiotensin-converting enzyme”, “endurance”, and “elite athletes” and individual sporting disciplines such as “running”, “cycling”, and “cross-country skiing” were used to identify the relevant literature. Additional studies were identified by searching for the reference lists of the studies eligible for full-text evaluation.

### 2.3. Selection Process

One reviewer (LS) initially assessed each study, and these were independently reviewed by other authors. As shown in Figure 1, after excluding articles whose titles did not involve the inclusion criteria, the remaining 93 abstracts were screened. After abstract evaluation, 31 studies underwent full-text evaluation, and 16 met the inclusion criteria and data requirements.

### 2.4. Data Extraction

Data were collected and recorded from the included studies by the same single reviewer (LS) and reviewed by SM. Records involved genotype distributions within endurance athletes, power athletes and controls, author and year of publication, population, sporting discipline, sample size, mean age, and female % for three categories (endurance athletes, power athletes, and controls).

### 2.5. Quality Assessment

Quality assessment was performed using a custom 10-point scale based on the Newcastle–Ottawa Scale (NOS) [31], aligned with the Strengthening the Reporting of Genetic Association Studies (STREGA) guidelines [32]. It included study design, case and control selection, laboratory methods, and HWE maintenance. All studies included in the final analyses had a minimum score of 6 (out of 10).

### 2.6. Statistical Analysis

Genotype data from individual studies were inputted into Metagenyo, an online genotype-focused meta-analysis package [33]. The following analyses were conducted: (1) endurance vs. control and (2) endurance vs. power. In addition, a subgroup analysis of different sports modalities was performed within the main analyses.

The endurance/control analysis included all studies. Some studies did not involve power athletes and, therefore, were excluded from endurance/power comparisons. Studies that involved a mixture of endurance sports were included in endurance/control and endurance/power analyses. However, if the sports were not analysed separately, these studies were excluded from the subgroup analysis by sporting discipline within both endurance/control and endurance/power comparisons.

This review chose recessive (II vs. ID + DD) and dominant (II + ID vs. DD) models to highlight the significance of the *ACE* I* allele, as it may confer an advantage even in heterozygotes. The models were deployed within forest plots, funnel plots, and subgroup analyses regarding sporting discipline. Significance was assessed using odds ratio, 95% confidence intervals, and *p*-values. Heterogeneity was tested using I^2^ because this statistic does not depend on the number of studies used in the meta-analysis, enabling easier comparisons [34]. Boundaries for heterogeneity were set based on the following guidance of Deeks et al. [35]: I^2^ 0–40% was low, 30–60% was moderate, 50–90% was substantial, and 75–100% was considerable. Finally, the risk of bias was assessed via funnel plot asymmetry using Egger’s test, whereby a *p*-value < 0.05 suggested publication bias was present.

## 3. Results

The collated data from various studies is presented in Table 1 (a and b). The genotype data (Table 1b) were used for meta-analysis and interpretations.

### 3.1. Meta-Analysis: Elite Endurance Compared to Healthy, Inactive Controls

**Figure 2:**
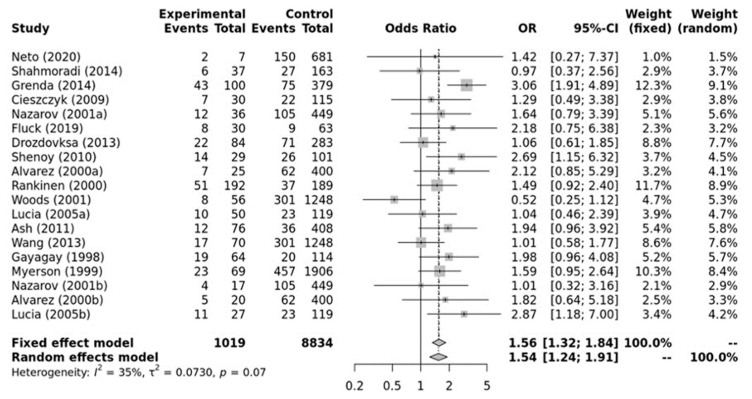
Forest plot of the genetic association comparing elite endurance athletes and controls using a recessive model of inheritance (II vs. ID + DD). Experimental: endurance athletes, Controls: healthy, inactive controls, OR: odds ratio, CI: confidence interval. References: Drozdovksa [6], Lucia [9], Myerson [13], Gayagay [36], Rankinen [37], Alvarez [38], Nazarov [39], Woods [40], Cieszczyk [41], Shenoy [42], Ash [43] Wang [44], Grenda [45], Shahmoradi [46], Fluck [47], Neto [48].

As shown in Figure 2, a significant association was found between the ACE II genotype and elite endurance athletes compared with the controls within the recessive model (OR, 1.54; 95%CI, 1.24–1.91). Moderate heterogeneity between studies was observed (*I*^2^ = 35%, *p*-value = 0.07). Additionally, no significant publication bias was observed (*p*-value = 0.650 for Egger’s Test) for this model.

**Figure 3:**
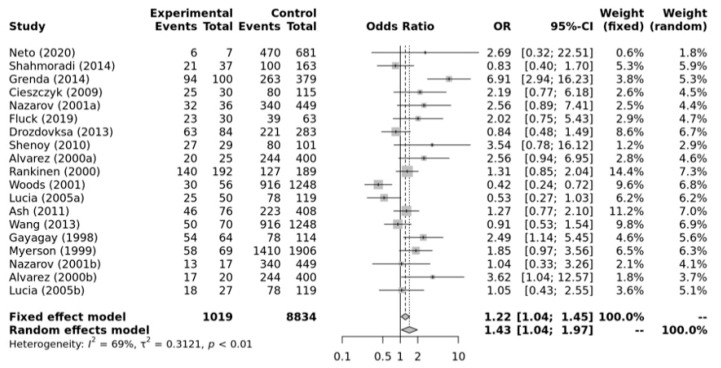
Forest plot of the genetic association comparing elite endurance athletes and controls using a dominant model of inheritance (II+ID vs. DD). Experimental: endurance athletes, Controls: healthy, inactive controls, OR: odds ratio, CI: confidence interval. References: Drozdovksa [6], Lucia [9], Myerson [13], Gayagay [36], Rankinen [37], Alvarez [38], Nazarov [39], Woods [40], Cieszczyk [41], Shenoy [42], Ash [43] Wang [44], Grenda [45], Shahmoradi [46], Fluck [47], Neto [48].

Figure 3 shows that there is a significant association between ACE II + ID genotypes and elite endurance athletes compared with the healthy inactive controls within the dominant model (OR, 1.43, 95%CI, 1.04–1.97). Substantial heterogeneity between studies (*I*^2^ = 69%, *p*-value < 0.01) and significant publication bias were observed (*p*-value = 0.029 for Egger’s Test) for this model. So, caution is warranted in these interpretations.

The subgroup analysis (Table 2) for different sport modalities showed significant associations for running and triathlon within the recessive (II vs. ID + DD) model (OR = 1.76; 95%CI = 1.26–2.47; *p*-value = 0.001 and OR = 2.69; 95%CI = 1.15–6.32, *p*-value = 0.023, respectively). Rowing and running showed significant associations within the dominant model (II + ID vs. DD) (OR = 2.38; 95%CI = 1.27–4.44; *p*-value = 0.007 and OR = 1.45; 95%CI = 1.04–2.02; *p*-value = 0.029, respectively). No associations were observed for cycling or swimming in either model.

### 3.2. Meta-Analysis: Elite Endurance Compared to Elite Power

**Figure 4:**
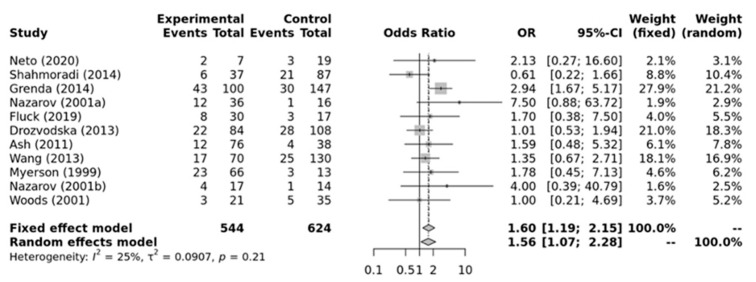
Forest plot of the genetic association comparing elite endurance and elite power athletes using a recessive model of inheritance (II vs. ID + DD). Experimental: endurance athletes, Controls: power athletes, OR: odds ratio, CI: confidence interval. References: Drozdovksa [6], Lucia [9], Myerson [13], Nazarov [39], Woods [40], Ash [43] Wang [44], Grenda [45], Shahmoradi [46], Fluck [47], Neto [48].

As shown in Figure 4, a significant association was found for the *ACE* II genotype and elite endurance athletes compared to ID+DD genotypes (OR = 1.56; 95%CI = 1.07–2.28) when compared against elite power athletes. Low heterogeneity between studies was observed (*I*^2^ = 25%, *p*-value = 0.21). Additionally, no significant publication bias was observed (*p*-value = 0.787 for Egger’s Test) for this model.

**Figure 5:**
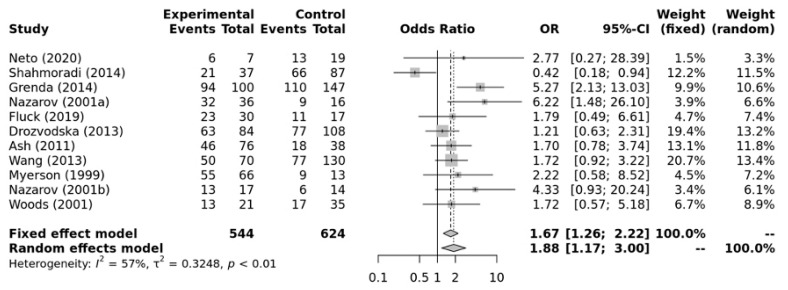
Forest plot of the genetic association comparing elite endurance and elite power athletes using a recessive model of inheritance (II + ID vs. DD). Experimental: endurance athletes, Controls: power athletes, OR: odds ratio, CI: confidence interval. References: Drozdovksa [6], Lucia [9], Myerson [13], Nazarov [39], Woods [40], Ash [43] Wang [44], Grenda [45], Shahmoradi [46], Fluck [47], Neto [48].

Figure 5 shows that there was a significant association between ACE II + ID genotypes and elite endurance athletes compared with the DD genotype (OR = 1.88, 95%CI = 1.17–3.00). Substantial heterogeneity between studies (*I*^2^ = 57%, *p*-value =< 0.01) was observed. No significant publication bias was observed (*p*-value = 0.199 for Egger’s Test) for this model.

The subgroup analysis (Table 3) for sporting disciplines showed significant associations for swimming within the recessive model (II vs. ID + DD) (OR = 2.27; 95%CI = 1.49–3.45; *p*-value = <0.001). Running and swimming were significantly associated within the dominant model (II + ID vs. DD) (OR = 2.45; 95%CI = 1.37–4.40; *p*-value = 0.003 and OR = 2.75; 95%CI = 1.71–4.41, *p*-value < 0.001, respectively). No associations were observed for cycling in either model.

## 4. Discussion

This systematic review and meta-analysis studied the association between elite endurance and the *ACE* I/D polymorphism by extracting data from 16 published studies. Elite endurance athletes were significantly associated with the II genotype compared with controls and elite power athletes. These results are broadly in line with the meta-analysis conducted by Ma et al. [14], who found a significant association with the *ACE* II genotype compared with the D allele carriage, specifically in endurance athletes compared with controls. Additionally, this review conducted a subgroup analysis regarding sporting discipline. Significant associations were explicitly observed in endurance runners, triathletes, rowers, and swimmers. There was no significant association between cyclists and *ACE* I/D polymorphism; however, this may have been due to a limited number of studies researching this discipline.

The subgroup analysis identified that the *ACE* II genotype was significantly associated with (1) endurance swimmers compared with power swimmers, (2) endurance runners compared with controls, and (3) triathletes compared with controls.

### 4.1. The I* Allele and Elite Endurance

The results support the hypothesis of this review that the *ACE* I* allele confers an advantage in elite endurance athletes. The modulation of the RAS characterises the underlying mechanism. The I* allele is associated with lower serum ACE activity, which prevents the degradation of bradykinin. In the heart, bradykinin mediates improved myocardial metabolic efficiency by increasing coronary flow and the conservation of glycogen and ATP stores during ischemia [16]. This may confer improvements in mitochondrial respiration efficiency and cardiac and skeletal muscle contractile function, therefore proving endurance performance [16].

Bradykinin increases the efficiency of skeletal muscle [49] by stimulating glucose extraction from the extracellular fluid [16], resulting in improved substrate delivery [39] and increased skeletal muscle glucose uptake [13,40]. This delays fatigue and allows for better conservation of energy stores [50]. Additionally, lower serum ACE activity reduces cardiac after-load for more efficient ventricular–vascular coupling during exercise [51].

Moreover, the *ACE* I* allele is associated with superior endurance training adaptations. Recent research has observed greater increases in the volume density of subsarcolemmal mitochondria and intramyocellular lipid stores as well as a higher cross-sectional area of embedded muscle fibres in *m.vastus lateralis* after endurance training compared with the DD genotype [52]. Flück et al. [47] observed higher sarcoplasmic, mitochondrial, and intramyocellular lipid volume densities in elite endurance cyclists and runners carrying the I* allele than those carrying the D* allele, observing a 79%, 54%, and 165% increase, respectively. These cellular factors underlie the enhanced aerobic capacity of elite endurance athletes, which the I* allele promotes.

The *ACE* I/D genotype may also confer mechanical/metabolic efficiency of skeletal muscle. A study of white male army recruits undergoing an 11-week training programme observed a significant increase in delta efficiency in II genotypes [53]. Delta efficiency is the amount of extra external power to the increase in metabolic power needed to overcome the extra external power [54]. This effect was seen alongside an increased relative anabolic response. Interestingly, before the training programme, subjects of different genotypes were phenotypically similar [53]. Although the study was conducted on army recruits and not elite endurance athletes, it does suggest that the *ACE* I* allele enhances the training response and may confer an advantage to the endurance phenotype via improvements in mechanical/metabolic efficiency, not just cardiorespiratory fitness. The mechanisms underlying this are speculative. It has been suggested that an increase in slow twitch muscle fibres is related to local increases in nitric oxide concentrations, which would help improve mitochondrial respiration through vasodilation and, therefore, skeletal muscle contractile function [53].

This research suggests that the *ACE* I/D polymorphism contributes to muscle specialisation via critical cellular hallmarks of muscle performance, which may impact training strategies to enhance sport-specific performance in elite endurance athletes [55].

### 4.2. The D* Allele and Elite Endurance

It is well established that the D* allele improves power performance by promoting a significant gain in strength and muscle volume after isometric strength training in quadricep muscles [56,57].

Combining II and ID showed significance in runners, swimmers, and rowers, suggesting that I* allele homozygotes are not required to observe an association with endurance. The D* allele may confer an advantage to endurance performance by improving power-oriented aspects. For instance, the outcome of most endurance events is heavily influenced by the athlete’s ability to conduct short bursts of maximal power, such as sprint finishes or breakaways. The D* allele confers a hypertrophic response to training by increasing serum ACE, resulting in increased ANG II levels, which stimulate endothelial, cardiac and smooth muscle cell growth [16]. As a result, the D* allele is conducive to an increase in blood pressure [41] as well as increased myocyte fibre size [13] and cardiac growth [36] via increased growth hormone release stimulated by ANG II [51]. This characteristic could aid endurance athletes by increasing glucose influx into the skeletal muscle for substrate utilisation, power output, and cardiac output, respectively. As a result, endurance athletes with the D allele would be able to reach and sustain the maximal intensities required for the power-oriented aspects of their disciplines. For instance, Lucia et al. [9] stated that the most elite participant in their study was the winner of a major 3-week cycling race. The cyclist had the DD genotype and won the competition based on his dominance of flat time trials because of his high muscle mass (weighing around 10kg heavier than the average endurance cyclist). The growth hormone gene neighbours the *ACE* gene, and some researchers have suggested possible linkage disequilibrium [16]. There is much scepticism around this [58]; however, a physiological interaction is highly likely due to the relationship between the ACE activity and growth hormone [39].

Conversely, the DD genotype may not confer an elite endurance phenotype because of its association with the inefficient import of serum glucose and disrupted mitochondrial metabolism [59] and lower transcription of lipid and glucose metabolism-associated factors in knee extensor muscle at the end of exhaustive exercise [60]. Overall, these effects would decrease the metabolic efficiency of the athlete, which is essential to endurance performance.

## 5. Potential Causes of Insignificant Associations

Contrary to the majority of studies, Ash et al. [43] found no significance between the *ACE* I/D polymorphism (rs4646994) and elite endurance runners; however, this is likely due to the use of elite Ethiopian athletes. Race influences *ACE* genotype distribution [13]; therefore, failure to observe an association may be due to the African bottleneck, which confers greater haplotype diversity across the *ACE* gene, but the *ACE* I/D (rs4646994) polymorphism does not influence serum ACE activity directly in African populations [44]. Conversely, the haplotype structure in European populations means that *ACE* I/D is in strong linkage disequilibrium with at least one functional site located in the 5′ region of the gene [44]. Alternatively, it may be that ACE activity is not heavily involved in endurance performance among Ethiopian athletes. Moreover, the lack of significant differences in the *ACE* I/D genotype between elite Ethiopian athletes and the general Ethiopian population may reflect natural genetic variation.

Rankinen et al. [37] found no association between *ACE* I/D and VO_2_ max; however, it is unlikely that the I* allele confers any enhancement via improvements in this parameter [40]. Furthermore, VO_2_ max values are not a given prerequisite for elite endurance performance, as endurance performance can vary greatly among individuals with an equal VO_2_ max [61,62]. Additionally, low values (<70 mL/kg/min) have been reported in world-class runners and cyclists [63,64].

Some studies failed to identify statistical significance between endurance and power athletes regarding the *ACE* I/D genotype [47]. However, this may have been because their sample size of elite athletes was relatively small for a gene association study. Studies that have deployed larger cohorts of elite athletes were more likely to identify significance [13,39].

Epigenetic regulation of the ACE gene may also explain inconsistencies in the effect of ACE I/D on the endurance phenotype [65]. It was reported that temporary methylation of the ACE gene could negate the rise in ACE activity caused by the D allele.

## 6. Strengths and Limitations

An inaccurate criterion for elite athlete status has limited other systematic reviews in this field, such as that by Ma et al. [14], by preventing significant associations from being observed. A clear criterion for elite standard endurance athletes strengthens this review. All included papers involved athletes of international standards; therefore, this review can confidently say that the results were not influenced by under-trained individuals. Genetic influences reported in moderately trained individuals may not translate to highly trained subjects because athletes have gained adaptive mechanisms by subjecting themselves to high volume and intensity from years of training and competition [47].

A limitation of this systematic review and meta-analysis is that the number of included studies is relatively small. The sub-optimal sample size is likely due to the high operational cost and difficulty accessing truly elite athletes [66] in the included studies. Additionally, only one study had data for triathletes; therefore, the significance of this association should be interpreted with caution.

## 7. Conclusions

Findings from this review indicate that the *ACE* I/D polymorphism is implicated in elite endurance performance and provides evidence to support the continued research of this gene variant. This review adds weight to the concept that there is a genetic component to obtaining elite status. It is important to note that the *ACE* I/D polymorphism should not be considered a “gene for human performance” but a marker of modulation that exerts effects on exercise performance only in the truly elite athlete population according to the nature of the sporting discipline. Possessing the optimal allele for different polymorphisms may be beneficial, but it is not critical. Because of the number of body systems that must interact, athletic performance is a very complex human trait [67], and the DNA polymorphisms identified thus far are not enough to determine athletic ability [8,68]. *ACE* I/D will likely have a more potent effect when other parameters of endurance performance are optimal and aligned.

## Figures and Tables

**Table 1:** (**a**). Studies included in this systematic review and meta-analysis. (**b**). Genotype data used in various meta-analysis models.

(a)														
Study ID	Background	Elite Endurance Athletes				Controls			Elite Power Athletes				Results	
Lead Author (Year)	Population	Sport	Size	Age (Mean/Range)	Female %	Size	Age (Mean/Range)	Female %	Sport	Size	Age (Mean/Range)	Female %	Summary Statistic (Endurance Athletes vs. Controls)	Summary Statistic (Endurance vs. Power)
Gayagay (1998) [36]	AUS	ROW	64	Not stated	33	114	<65	34	N/A				I allele increased in athletes (*p* < 0.02)	N/A
Myerson (1999) [13]	GBR	RUN	71	Not stated	Not stated	1906	Not stated	Not stated	RUN	20	Not stated	Not stated	I allele increased white athletes (*p* = 0.039)	I allele increased with distance run (*p* = 0.009)
Rankinen (2000) [37]	CAN/DEU/FIN/USA	XC-SKI BITHLN NC RUN CYC	192	Not stated	0	189	Not stated	0	N/A				No significant differences	N/A
Alvarez (2000) [38]	ESP	CYC RUN	45	21–35	0	400	<55	38	N/A				Excess of II/ID genotypes in athletes (*p* = 0.0008)	N/A
Nazarov (2001) [39]	RUS	SWIM RUN XC-SKI TRITHLN	111	Not stated	Not stated	449	18–45	40	SWIM RUN	30	Not stated	Not stated	No significant differences	I allele increased with event duration (*p* = 0.032)
Woods (2001) [40]	Not stated	SWIM	21	Not stated	Not stated	1248	19.7 ± 2.5	Not stated	SWIM	35	Not stated	Not stated	No significant differences	D allele increased in short distance athletes
Lucia (2005) [9]	ESP	CYC RUN	77	26–27	0	119	42 ± 12	0	N/A				Excess of DD genotype in cyclists (*p* < 0.05) and II genotype in runners (*p* < 0.05)	N/A
Cieszczyk (2009) [41]	POL	ROW	30	Not stated	0	115	19–23	0	N/A				I allele increased in athletes (*p* = 0.038)	N/A
Shenoy (2010) [42]	IND	TRITHLN	29	20–25	0	101	20–25	Not stated	N/A				I allele increased in athletes (*p* = 0.02)	N/A
Ash (2011) [43]	ETH	RUN	76	Not stated	42	410	Not stated	11	RUN THROW JUMP	38	Not stated	53	No significant differences	No significant differences
Drozdovska (2013) [6]	UKR	XC-SKI ROW	84	Not stated	Not stated	283	14–54	43	RUN SWIM JUMP THROW	108	Not stated	Not stated	No significant differences	No significant differences
Wang (2013) [44]	EUR/CMNWLTH/USA/RUS/JPN/TWN	SWIM	230	Not stated	32	2492	Not stated	Not stated	SWIM	296	Not stated	41	No significant differences	N/A
Grenda (2014) [45]	POL	SWIM	49	20.3 ± 2.7	51	379	22.6	41	SWIM	147	20.3 ± 2.7	46	I allele increased in athletes	ID (*p* = 0.009) and II (*p* = 0.0002) genotypes increased in endurance
Shahmoradi (2014) [46]	IRN	CYC	37	18–40	0	163	18–40	0	WTLIFT RUN JUMP THROW	87	18–40	0	I allele increased in athletes (0.03)	Increased D allele in endurance (*p* = 0.045)
Fluck (2019) [47]	Not stated	CYC RUN	30	30.1 ± 5.7	Not stated	63	29.5	Not stated	SHTPUT WTLIFT	17	25.4 ± 10.1	Not stated	No significant differences	No significant differences
Neto (2022) [48]	BRA	SWIM	7	19–30	Not stated	681	Not stated	Not stated	SWIM	19	18–20	Not stated	Lower frequency of DD genotypes (*p* = 0.006)	Lower frequency of DD genotypes (*p* = 0.004)
**(b)**														
**Endurance vs. Controls**														
**Study**		**Discipline**		**II_Athlete**		**ID_Athlete**			**DD_Athlete**		**II_Control**		**ID_Control**	**DD_Control**
Neto (2022) [48]		Swimming		2		4			1		150		320	211
Shahmoradi (2014) [46]		Cycling		6		15			16		27		73	63
Grenda (2014) [45]		Swimming		43		51			6		75		188	116
Cieszczyk (2009) [41]		Rowing		7		18			5		22		58	35
Nazarov (2001) [39]		Swimming		12		20			4		105		235	109
Fluck (2019) [47]		Mixed		8		15			7		9		30	24
Drozdovksa (2013) [6]		Mixed		22		41			21		71		150	62
Shenoy (2010) [42]		Triathlon		14		13			2		26		54	21
Alvarez (2000) [38]		Cycling		7		13			5		62		182	156
Rankinen (2000) [37]		Mixed		51		89			52		37		90	62
Woods (2001) [40]		Swimming		8		22			26		301		615	332
Lucia (2005) [9]		Cycling		10		15			25		23		55	41
Ash (2011) [43]		Running		12		34			30		36		187	185
Wang (2013) [44]		Swimming		17		33			20		301		615	332
Gayagay (1998) [36]		Rowing		19		35			10		20		58	36
Myerson (1999) [13]		Running		23		35			13		457		953	496
Nazarov (2001) [39]		Running		4		9			4		105		235	109
Alvarez (2000) [38]		Running		5		12			3		62		182	156
Lucia (2005) [9]		Running		11		7			9		23		55	41
**Endurance vs. Power**														
**Study**		**Discipline**		**II_Endurance**		**ID_Endurance**			**DD_Endurance**		**II_Power**		**ID_Power**	**DD_Power**
Neto (2022) [48]		Swimming		2		4			1		3		10	6
Shahmoradi (2014) [46]		Cycling		6		15			16		21		45	21
Grenda (2014) [45]		Swimming		43		51			6		30		80	37
Nazarov (2001) [39]		Swimming		12		20			4		1		8	7
Fluck (2019) [47]		Mixed		8		15			7		3		8	6
Drozdovksa (2013) [6]		Mixed		22		41			21		28		49	31
Ash (2011) [43]		Running		12		34			30		4		14	20
Wang (2013) [44]		Swimming		17		33			20		25		52	53
Myerson (1999) [13]		Running		23		35			13		3		8	9
Nazarov (2001) [39]		Running		4		9			4		1		5	8
Woods (2001) [40]		Swimming		3		10			8		5		12	18

Abbreviations: N/A: Not Available, ROW: rowing, RUN = running, XC-SKI = cross-country skiing, BITHLN = biathlon, TRITHLN = triathlon, NC = Nordic combined, CYC = cycling, and SWIM = swimming.

**Table 2:** Subgroup analysis showing the associations between the *ACE* I/D polymorphism and specific sporting disciplines when compared against healthy, inactive controls using a recessive and dominant model.

Model	Discipline.	Number of Studies	OR	95%CI	*p*-Value
Recessive model (II vs. ID+DD)					
	Cycling	3	1.28	0.76–2.16	0.342
	Rowing	2	1.69	0.95–3.03	0.072
	Running	5	1.73	1.23–2.42	0.001
	Swimming	5	1.33	0.67–2.62	0.413
	Triathlon	1	2.69	1.15–6.32	0.023
Dominant model (II+ID vs. DD)					
	Cycling	3	0.96	0.42–2.20	0.930
	Rowing	2	2.38	1.27–4.44	0.007
	Running	5	1.39	1.00–1.93	0.047
	Swimming	5	1.64	0.57–4.72	0.358
	Triathlon	1	3.54	0.78–16.12	0.102

**Table 3:** Subgroup analysis showing the associations between the ACE I/D polymorphism and specific sporting disciplines when compared against elite power athletes using a recessive and dominant model.

Model	Discipline.	Number of Studies	OR	95%CI	*p*-Value
Recessive model (II vs. ID+DD)					
	Cycling	1	0.61	0.22–1.66	0.331
	Running	3	2.21	0.96–5.09	0.061
	Swimming	5	2.27	1.49–3.45	<0.001
Dominant model (II+ID vs. DD)					
	Cycling	1	0.42	0.18–0.94	0.036
	Running	3	2.45	1.37–4.40	0.003
	Swimming	5	2.75	1.71–4.41	<0.001

## Data Availability

All data used in this study are included in the manuscript itself.
